# Nutrition and Outcome of 100 Endoscopic Gastrostomy-Fed Citizens with Severe Dementia

**DOI:** 10.3390/nu15122753

**Published:** 2023-06-15

**Authors:** Diogo Sousa-Catita, Paulo Mascarenhas, Cátia Oliveira, Miguel Grunho, Carla Adriana Santos, Jorge Fonseca

**Affiliations:** 1 Grupo de Patologia Médica, Nutrição e Estudos Clínicos (PaMNEC) of Egas Moniz Center for Interdisciplinary Research (CiiEM), Egas Moniz School of Health & Science, Caparica, 2829-511 Almada, Portugal; pmascarenhas@egasmoniz.edu.pt (P.M.); miguelgrunho@gmail.com (M.G.); jorgedafonseca@gmail.com (J.F.); 2 GENE—Artificial Feeding Team, Gastroenterology Department, Hospital Garcia de Orta, 2805-267 Almada, Portugal; sofi.doliveira@gmail.com (C.O.); carla.adriana.santos@hotmail.com (C.A.S.); 3 Residências Montepio—Serviços de Saúde, SA—Rua Julieta Ferrão N° 10–5°, 1600-131 Lisboa, Portugal; 4 Neurology Department, Hospital Garcia de Orta, 2805-267 Almada, Portugal

**Keywords:** severe dementia, nutritional status, percutaneous endoscopic gastrostomy

## Abstract

Dementia is a rising public health concern. Feeding and nutritional problems increase as the disease progresses, affecting the clinical course and caregiver burden. While some guidelines advise against percutaneous endoscopic gastrostomy (PEG) and tube feeding in advanced dementia, conflicting evidence exists. This study aims to evaluate the nutritional status and influence of PEG feeding on the outcome and evolution of nutritional/prognosis markers of patients with severe dementia (PWSD) who underwent gastrostomy for nutritional support. We conducted a 16-year retrospective study on 100 PEG-fed PWSD with strong familial support. We evaluated the survival PEG-feeding period, safety, and objective nutritional/prognosis data on the gastrostomy day and after 3 months: Body Mass Index (BMI), Mid Upper Arm Circumference, Tricipital Skinfold, Mid-Arm Muscle Circumference, albumin, transferrin, total cholesterol, and hemoglobin. Most patients presented low values in these nutritional/prognosis parameters. No major life-threatening PEG complications were reported. The mean survival time after gastrostomy was 27.9 months (median of 17 months). Female sex, BMI recovery at 3 months, and higher baseline hemoglobin levels were associated with a reduced risk of death and increased survival time. The study concluded that, in carefully selected PWSD with strong familial support, PEG feeding can improve nutritional status and have a positive impact on survival.

## 1. Introduction

Dementia is an umbrella term for acquired, chronic, progressive, age-related, and functionally impairing neurocognitive decline, encompassing several different and heterogeneous clinical conditions including, most commonly, Alzheimer’s disease, vascular dementia, Lewy body disease, and frontotemporal dementia [1]. Dementia is recognized as a burdensome public health issue, both currently and in the decades to come [2]. Approximately 47 million people worldwide have dementia, which is predicted to rise to a staggering 132 million by 2050 [3]. Various clinical dementia staging scales have been developed throughout the years, each with different pros, cons, dissemination, and implementation, including the widely used Clinical Dementia Rating (CDR), Global Deterioration scale (GDS), and Functional Assessment Staging (FAST) [4,5,6,7].

Nutritional and feeding problems are known to occur throughout the illness, across its different stages, becoming more common as the disease progresses, and negatively impacting the clinical course, outcome, and caregiver burden [8]. Therefore, it is unsurprising that several studies have focused on the nutritional issues of patients with advanced dementia. The use of enteral tube feeding in individuals with severe dementia has been the subject of several studies and systematic reviews [9,10,11,12,13] and was addressed in the most recent clinical practice guidelines covering this issue, promoted by the American Geriatric Society [14] and the European Society for Clinical Nutrition and Metabolism (ESPEN) [4]. Both societies advise against the use of enteral tube feeding in these patients (while advocating for careful hand-feeding instead), after assuming that it is not associated with longer survival or improvement in nutritional status, that it causes the excessive use of restraints, and that it is not effective in preventing pressure ulcers and aspiration pneumonia [4,14]. Nevertheless, some authors expressed concern regarding the quality of the scientific evidence used to formulate the recommendations, arguing that it suffers from bias and inaccurate methodologies, including an inadequate control group and a lack of data on quality of life [15]. Most of the studies that are used to advocate against tube feeding report short survival periods, with or without enteral tube feeding [10], in contrast with the more extended survival period of PEG-fed dementia patients seen during the clinical practice of several teams, including our artificial nutrition team. In fact, PEG feeding has long been advised for dementia patients by teams taking care of those persons [16,17,18].

Additionally, recent data suggest that if there is a medical indication for enteral tube feeding, it should not, a priori, be precluded just because the patient has a dementia diagnosis [19]. These authors call for a critical revision of the recommendations on enteral tube feeding in patients with advanced dementia and uphold, for the time being, that the decision-making process should be personalized as much as possible, avoiding generalizations [15]. This conflict of opinions continues to fuel the ongoing controversy regarding whether one should continue hand feeding or initiate enteral tube feeding in this population.

Overall, acknowledging the gaps in research in this field, the complex ethical dilemmas, and the heterogeneity within dementia syndromes, one should bear in mind that a “one-size-fits-all” approach may not be suitable for the management of nutritional and feeding problems of citizens with advanced dementia.

Our artificial nutrition team, GENE (Grupo de Estudo de Nutrição Entérica/parentérica), evaluated every patient with dementia proposed to undergo endoscopic gastrostomy for long-term tube feeding. People with severe dementia also underwent endoscopic gastrostomy if they maintained a close relationship with family, friends, and caregivers, and if a long survival period was expected.

The current study aims to demonstrate that the PEG procedure is safe for “Persons with Severe Dementia” (PWSD) criteria and may contribute to a better nutritional status of PWSD.

### Objectives

1. To evaluate PWSDs’ clinical and nutritional status at three GENE routine evaluation follow-ups. T0—at the day of the endoscopic gastrostomy procedure, T1—1 month after gastrostomy, and T2—three months after gastrostomy.
  Using several easily accessible tools, even with patients who have speech difficulties, namely:
  1.1 Anthropometry.
  1.2 Laboratory data.

2. To evaluate the survival of PEG-fed PWSD after the gastrostomy procedure.
3. To evaluate the impact of nutritional status on the survival outcome of PWSD patients that underwent endoscopic gastrostomy, using anthropometric and biochemical markers.
4. To evaluate the impact of PEG feeding on the nutritional status and patients’ outcomes, evaluated using anthropometric and biochemical markers.
5. To evaluate the occurrence of major complications from the gastrostomy procedure or PEG-feeding to establish the safety of endoscopic gastrostomy on PWSD.

## 2. Materials and Methods

### 2.1. Patients

We studied consecutive adult patients with severe dementia, in accordance with the standards of the International Classification of Diseases (ICD) (ICD-9 from 2005 to 2016, and ICD-10 from 2017 onwards), who were referred and underwent endoscopic gastrostomy to have PEG nutritional support, for 16 years, from January 2005 to December 2020. Patients were considered eligible if they were referred to PEG as severe dementia by their attending clinicians, whatever dementia staging tool was used. All data are part of the routine evaluation of PEG patients and were collected from GENE clinical files.

All dementia patients in our artificial feeding team files were eligible for the study. The exclusion criteria were:

1. Early dementia stage.
2. Insufficient data in the clinical file.

### 2.2. Safety

Complications with PEG were a significant concern and our team aimed to ensure this procedure’s safety. During the follow-ups, we recorded and evaluated all potential major complications associated with PEG.

### 2.3. Clinical Outcome

We collected the survival period (in months) of the PEG dementia patients from the endoscopic gastrostomy procedure until death or until 31 December 2020.

### 2.4. Anthropometric Evaluation

We recorded clinic and anthropometric data on the day of the endoscopic gastrostomy or the day before (T0), one month after endoscopic gastrostomy (T1), and three months after endoscopic gastrostomy (T2). The anthropometry measurements followed the International Society for the Advancement of Kinanthropometry manual. We obtained three consecutive measurements each time. The clinical file record represents those three measurements’ mean.

Body mass index (BMI): BMI was obtained in most patients using the equation Weight/Height2. If patients were bedridden and could not stand up for weight and height evaluation, BMI was estimated using the mid-upper arm circumference (MUAC) and regression equations described by Powell-Tuck and Hennessy [20]; this method has been previously used and proved to provide a reliable BMI estimation in PEG patients [21,22]. Each patient was classified by the WHO classification according to their age [23] (Table 1).

Mid Upper Arm Circumference (MUAC) was evaluated using an inextensible measuring tape with a 1 mm resolution. MUAC results from evaluating several tissues represent fat and lean mass.

Tricipital skinfold (TSF) was measured using a Lange Skinfold caliper with a 1 mm resolution. TSF evaluates the subcutaneous adipose tissue and estimates adipose reserves.

The Mid-Arm Muscle Circumference (MAMC) was calculated according to the equation: MAMC = MUAC (cm) − 0.314 × TSF (mm). The MAMC allows us to estimate lean and muscle mass.

For each patient, MUAC, MAMC, and TSF were compared with reference values of the National Health and Nutrition Examination Survey (NHANES) through the comparison with the Frisancho reference tables [24,25].

Although nutritional evaluation could benefit from sophisticated devices for measuring body composition, such as bioelectrical impedance analysis (BIA) or CT Scan analysis, those devices were not available for all patients. Although less precise, BMI and anthropometry measures are inexpensive and widespread nutritional evaluation tools, classically used to evaluate fat/lean mass [26] and available everywhere, even in institutions with scarce resources.

### 2.5. Laboratory Evaluation

A blood sample was obtained minutes before the endoscopic gastrostomy procedure (T0) and one and three months after the gastrostomy procedure (T1/T2). Blood samples were obtained between 8:00 and 10:00 a.m. following at least 12 h of fasting. Serum Albumin < 3.5 g/dL, serum Transferrin < 200 mg/dL, serum Total Cholesterol < 160 mg/dL, and Hemoglobin (Male < 13 g/dL, Female < 12 g/dL) were considered low values, suggestive of poor prognosis and/or malnutrition [27,28,29]. Nevertheless, laboratory data were always regarded as dependent on several non-nutritional influences.

### 2.6. Ethical Considerations

All subjects, relatives, caregivers, and responsible persons were informed of the procedures of the Artificial Feeding Team for PEG-feeding patients, and relatives and responsible persons gave their informed consent. This retrospective study was approved by the Hospital Garcia de Orta Ethics Committee.

### 2.7. Statistics

We used the SPSS software version 25 (Armonk, NY, USA: IBM corporation) to compute the descriptive statistics and perform survival analysis. Survival analysis included plotting the Kaplan–Meier patients’ survival rate curves, estimating the mean and the median survival time, and evaluating the dependency on survival time of predictor variables by modelling the Cox proportional-hazards regression model. Patient death was the event of interest in the model. Censored data were defined as coming from patients who were alive at the study’s end. The Walt test evaluated the linear combination of the Cox model estimated parameters, and the Breslo–Day statistic tested for the hazard ratios homogeneity between category variables levels.

## 3. Results

### 3.1. Subjects

This study involved 120 patients with the diagnosis of dementia, in accordance with the standards of the International Classification of Diseases (ICD) in effect at the time of the referral (ICD-9 from 2005 to 2016, and ICD-10 from 2017 onwards), at an advanced stage. Of these patients, 20 were excluded for incomplete data. The remaining 100 patients presented all data accessible on clinical records, except Hemoglobin, which was only available in 65. Of these 100 patients displaying all the criteria, 39 were males and 61 were females. Ages ranged from 51 to 100 years (mean: 78.4 years.; median: 80.5 years.). Most patients (*n* = 88) were older citizens, at 65 years old or older. Only 12 were younger adults, at less than 65 years old. Table 2 displays the characterization of subjects’ anthropometry and laboratory serum data.

### 3.2. Anthropometry at T0

BMI was obtained in all 100 patients. For 46 patients, BMI was estimated using the Powell–Tuck and Hennessy regression equations. BMI ranged from 14 kg/m^2^ to 41 kg/m^2^ (mean: 23.71 kg/m^2^; median: 22.80 kg/m^2^). The WHO classification was used according to age. Subscribing to this classification, 39 patients displayed low BMI. At ages lower than 65, 2 patients displayed low BMI (<18.5 kg/m^2^), and with an age of 65 or more, 37 patients displayed a low BMI of <22 kg/m^2^ (Table 2).

Compared with Frisancho criteria [24], 77 showed MUAC in the low range, 84 displayed low TSF, and 52 patients showed MAMC in the low range.

### 3.3. Laboratory Assessment at T0

On the day of gastrostomy, 64 patients presented low serum Albumin, 62 showed low serum Transferrin, 51 displayed low serum Total Cholesterol, and 43 out of 65 displayed low Hemoglobin.

### 3.4. Safety

During the follow-ups, the detection of problems associated with PEG tube placement was considered. From all dementia patients, no major life-threatening problems were reported or detected.

### 3.5. Evolution Data According to Follow-Ups (T0 to T2)

Only BMI increased during follow-ups (T0 to T2). Hemoglobin, Albumin, and Transferrin decreased during follow-ups (T0 to T2). However, the only significant change was in Albumin and Transferrin. These two laboratory parameters significantly decreased at the 3-month evaluation (*p* = 0.001). Total Cholesterol increased during the first month, however, without statistical significance (Table 3).

### 3.6. Clinical Outcome

In December 2020, from the 100 patients who fulfilled the included criteria, 11 were still alive, and all alive patients were still PEG-fed and followed up at the Artificial Nutrition Outpatients Clinic.

### 3.7. Kaplan–Meier Survival Analysis

**Figure 1:**
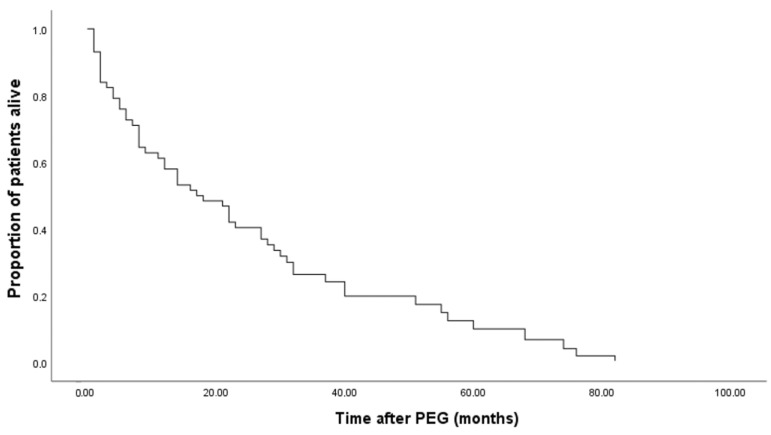
Kaplan–Meier curve of cumulative survival in PWSD.

**Figure 2:**
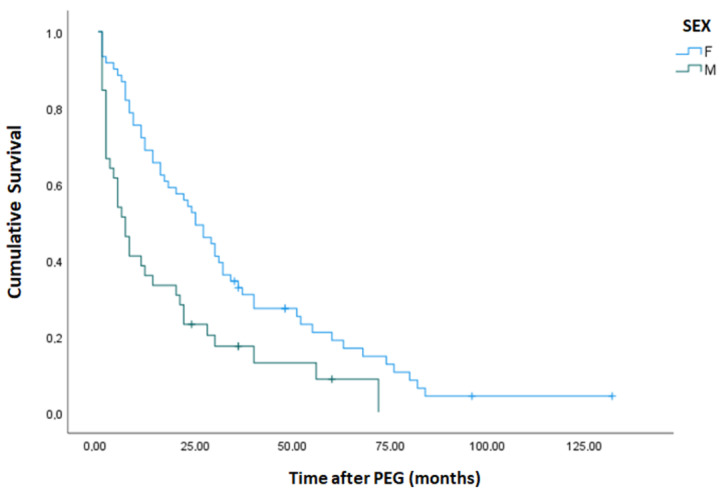
Kaplan–Meier curve of cumulative survival by gender in PWSD.

Of 100 patients, 10 deceased during the first month (6 Males and 4 females), and 9 deceased between the first and the third months (8 males and 1 female). Therefore, after the first three months, only 19 patients were deceased, and 81 were alive and PEG-fed (Kaplan–Meier Curves in Figure 1 and Figure 2).

The mean survival of all patients after the gastrostomy was almost 28 months, while the median was 17 months. In the female group, the mean survival time was significantly higher than in the male group (Table 4; *p* < 0.001, Breslow–Day test).

On average, those with low BMI at T0 were the ones who died first, and those with normal/high BMI at T0 were the ones who survived longer; however, these differences were not significant (*p* = 0.716, Breslow–Day test) (Table 5).

### 3.8. Survival According to Data Follow-Ups (T0 to T2)

Our Cox regression results show that being female (*p* = 0.012), having positive BMI recovery during PEG feeding (*p* = 0.023), and having higher baseline Hemoglobin (*p* = 0.005) had a significant effect on reducing the risk of death, leading to an increase in survival. However, baseline Hemoglobin shows the weakest effect, with one more unit of Hemoglobin associated with an average 2% decrease in death risk (Table 6). Therefore, considering the modifiable factors, BMI recovery at three months was the most important factor for better survival (Table 6). For each point more in BMI recovery, there was a 10% significant reduction in the risk of experiencing death (*p* = 0.023) (Table 6). In arm anthropometry, MUAC and MAMC positive recoveries, evaluated at the end of 3 months, were both associated with longer survival times (*p* = 0.172 and *p* = 0.094, respectively) (Table 6). In fact, MAMC *p* = 0.09 represents a statistical trend that deserves clinical appraisal.

## 4. Discussion

Tube feeding in patients with severe dementia is a controversial issue. Some studies and guidelines recommend avoiding tube feeding use in patients with severe dementia because no clear evidence demonstrates the benefits of PEG feeding in nutritional status and survival time. The studies supporting ESPEN guidelines on nutrition in dementia [8] used patients in different conditions and institutions, such as patients with stroke [30], and this pathology may have a lower survival time than neurodegenerative disorders. In other studies, the findings were inconclusive regarding whether enteral tube feeding benefits dementia patients, likely due to US patients being different when compared to their European counterparts [9]. In contrast, PWSDs have good medical, nutritional, and family support in our social reality. These three points are the major base for moving towards PEG placement.

Despite advanced dementia criteria, some patients have a strong family relationship that justifies, in ethical terms, the extension of life, likely giving greater comfort and improving the nutritional status.

Our patients with severe dementia had poor oral intake, which translated into a nutritional risk. Therefore, our multidisciplinary team discussed the benefits and disadvantages of different approaches with patients’ families. All patients also had an estimated survival longer than one month at admission; this is suitable for generic PEG placement in patients who do not resume oral feeding according to recommendations [31].

The present study’s patients’ average age is 78, so most of our dementia cases are linked to age [32]. The mean survival of our patients was more than 27 months (median of 17 months). The decreased values we found for the medians were due to the presence of censored cases that inflated the survival time mean values. The greatest differences between mean and median survival time are observed in the Normal BMI group due to this group including the major proportion of censored cases. Only 19% of patients died during the first 3 months of PEG feeding. As demonstrated in our previous study [18], these interesting results reinforce the need to feed those patients via PEG. We suspect that the mean may be influenced by a group of patients with greater longevity (including those still alive). However, a 17-month median is much higher than the survival of the patients described in the studies that support the guidelines. We believe some previous studies focused on terminal patients, not the general population of PWSD with advanced disease criteria, who may have a much longer life span.

In this study, we were concerned during the follow-ups about checking for complications related to the PEG. We were careful to explain to caregivers all the precautions they should take to minimize the risk of any complications associated with PEG. In this way, major complications associated with PEG were not detected or reported in our patients.

At admission, we evaluated the BMI in all patients, a worldwide anthropometric parameter validated to assess nutritional status. In our study, mean BMI increased during the two evaluations but not significantly. The three months were likely too short to display a more significant BMI improvement, but this is the standard for follow-up appointments. However, individual BMI could improve survival by reducing the death risk by 10% for each point increase in BMI at the end of 3 months. This is a clear demonstration that PEG feeding in these carefully selected patients with severe dementia can improve nutritional status, and this has a positive impact on survival.

Unlike BMI, Albumin and Transferrin displayed a decrease during the first three months. Since several factors may be involved, this decrease is likely linked to inflammatory, non-nutritional factors. Nevertheless, these laboratory data do not show any impact on the survival of our patients.

Most anthropometric data displayed levels related to poor nutrition, so PEG placement could be a tool for improvement since nutritional support could be better than oral intake only. Arm anthropometry (MUAC, TSF, and MAMC) data showed malnutrition in over eighty per cent of the patients. The estimation of fat and fat-free reserves also revealed poor nutritional status. TSF recognized more malnourished patients than MAMC, which suggests that fat tissue is more depleted and muscle mass is more preserved in our patients’ conditions. Moreover, MAMC is an independent outcome predictor, highlighting the importance of lean mass in patient survival, likely reflecting more prolonged survival compared to other studies, and may be linked to better health and family care. MUAC and MAMC showed a possible influence on survival since for every point increased at the end of three months, there was a lower risk of death by approximately 9 and 12%, respectively. Most anthropometric data improved significantly (even without statistical significance) over time and gradually reduced as the disease progressed. Interestingly, MAMC shows an increase over the months of PEG feeding, with a trend of significance (*p* = 0.094), and increasing MAMC reduces the risk of death, highlighting the importance of PEG feeding for recovering lean body mass and improving survival.

BMI, Hemoglobin, and female gender were the only three parameters with statistical significance on survival obtained at the beginning of PEG feeding on the day of the gastrostomy procedure. In our experience, the female gender is generally associated with slightly better nutritional status at the beginning of PEG feeding. We empirically believe this is linked to cultural factors favoring earlier acceptance of PEG tube placing in women more than men. We have seen a trend of early acceptance of PEG and better initial nutritional status in women and in PEG patients with other neurological disorders or with head or neck cancer. Our team believes that this reflects sociological differences in attitudes between genders, with males being less likely to accept PEG.

Our study has some limitations. One is the nonexistence of a control group to compare our results. We chose patients with good family support and believe that having a control group in this situation is unsuitable and unethical. Some missing data also limited our results. We completed processing patient data in December 2020 due to the COVID-19 pandemic. Several patients did not continue their follow-up (refusing to go to the hospital), and some records were incomplete. Moreover, a wider group could allow for more solid evidence, but these carefully selected PWSDs are scarce, and multicenter studies would be necessary for enrolling a larger group.

## 5. Conclusions

Our team selected PWSD with strong family support and adequate medical and nutritional care, which differs from PWSD patients with a terminal condition in several studies. We demonstrate that, in these selected patients, PEG is a safe procedure, and PEG feeding can improve anthropometric data, leading to more prolonged survival. Female gender, baseline Hemoglobin, and BMI, as well as BMI improvement at the end of 3 months, were markers for better outcomes. One unit of increase in BMI recovery was associated with a 10% decrease in death risk. In our study, mean survival of 27 months (median of 17 months) was better than most studies and likely reflects the benefit of a gastrostomy in PWSD. Based on our results, we recommend that PEG should be considered in patients with severe dementia with strong family support when risk factors related to malnutrition are present.

## Figures and Tables

**Table 1:** Body mass index (BMI) classification according to age.

	Low	Normal	High
<65 Years	<18.5 kg/m^2^	≥18.5–<25 kg/m^2^	≥25 kg/m^2^
≥65 Years	<22 kg/m^2^	≥22–<27 kg/m^2^	≥27 kg/m^2^

**Table 2:** Characterization of subjects by anthropometry and laboratory serum data.

	Total (*n* = 100)	Male (*n* = 39)	Female (*n* = 61)	Total Mean
Anthropometry Results				
BMI	39 Low (39%)	13 Low (33.3%)	26 Low (42.6%)	
	33 Normal (33%)	16 Normal (41%)	17 Normal (27.8%)	
	28 High (28%)	10 High (25.7%)	18 High (29.6%)	
MUAC	77 Low (77%)	31 Low (79.5%)	46 Low (75.4%)	
	23 Normal (23%)	8 Normal (20.5%)	15 Normal (24.6%)	
TSF	84 Low (84%)	29 Low (74.4%)	55 Low (90.1%)	
	16 Normal (16%)	10 Normal (25.6%)	6 Normal (9.9%)	
MAMC	52 Low (52%)	31 Low (79.5%)	21 Low (34.4%)	
	48 Normal (48%)	8 Normal (20.5%)	40 Normal (65.6%)	
Laboratory serum data				
Albumin	64 Low (64%)	31 Low (79.5%)	33 Low (54.1%)	3.25 g/dL
	36 Normal (36%)	8 Normal (20.5%)	28 Normal (45.9%)	
Transferrin	62 Low (62%)	35 Low (89.7%)	45 Low (73.7%)	170.6 mg/dL
	34 Normal (34%)	4 Normal (10.3%)	16 Normal (26.3%)	
Total Cholesterol	51 Low (51%)	28 Low (71.8%)	23 Low (33.7%)	164.4 mg/dL
	49 Normal (49%)	11 Normal (28.2%)	38 Normal (66.3%)	
Hemoglobin * (*n* = 65)	43 low (66.1%)	23 Low (88.5%)	20 Low (50%)	11.3 g/dL
	22 Normal (33.9%	2 Normal (11.5%)	20 Normal (50%)	

BMI—Body mass index; BMI classification according to age, <65 y, low BMI is <18.5 kg/m^2^, normal BMI is between 18.5 kg/m^2^ and <25 kg/m^2^, and high BMI is ≥25 kg/m^2^; ≥65 y, low BMI is <22 kg/m^2^, normal BMI is between 22 kg/m^2^ and <27 kg/m^2^, and high BMI is ≥27 kg/m^2^; (MUAC)—mid-upper arm circumference <90% low, ≥90–110% normal; (TSF)—tricipital skinfold results, <90% low, ≥90–110% normal and (MAMC)—mid-arm muscle circumference <90% low, ≥90–110% normal; Albumin < 3.5 g/dL (low), Transferrin < 200 mg/dL (low), Total cholesterol < 160 mg/dL (low); Hemoglobin—Male 14–18 g/dL (normal), Female 12–16 g/dL (normal). * Only 65 patients were evaluated for Hemoglobin.

**Table 3:** Differences between follow-ups relative to all data.

	Comparisons Pairwise	Recovery [95% Conf. Int.]	Sig.
BMI	1/2	−0.328 [−1.468, 0.812]	0.566
	2/3	0.073 [−0.496, 0.642]	0.798
	1/3	−0.255 [−1.494, 0.984]	0.681
MUAC	1/2	2.172 [−5.473, 9.817]	0.571
	2/3	0.023 [−0.498, 0.545]	0.929
	1/3	2.195 [−5.544, 9.934]	0.572
MAMC	1/2	−0.666 [−8.317, 6.984]	0.862
	2/3	0.023 [−0.498, 0.545]	0.929
	1/3	−0.643 [−8.384, 7.098]	0.868
TSF	1/2	0.468 [−0.226, 1.162]	0.182
	2/3	−0.026 [−0.751, 0.700]	0.944
	1/3	0.442 [−0.438–1.323]	0.318
Albumin	**1/2**	**−0.191 [−0.324, −0.059]**	**0.006**
	2/3	−0.124 [−0.250, 0.001]	0.052
	**1/3**	**−0.316 [−0.490, −0.141]**	**0.001**
Transferrin	1/2	−14.356 [−29.126, 0.413]	0.056
	**2/3**	**−18.375 [−31.473, −5.277]**	**0.007**
	**1/3**	**−32.731 [−51.665, −13.797]**	**0.001**
Total Cholesterol	1/2	−4.707 [−14.319, 4.904]	0.328
	2/3	2.976 [−9.252, 15.203]	0.626
	1/3	−1.732 [−17.538, 14.075]	0.826
Hemoglobin	1/2	4.454 [−8.045, 16.953]	0.467
	2/3	−9.177 [−20.962, 2.609]	0.120
	1/3	−4.723 [−12.191, 2.745]	0.203

Recovery with statistical significance (<0.05) is highlighted in bold.

**Table 4:** Means and medians by gender for survival time.

Survival (Month)	Mean ^a^	Mean CI 95%	Median
Female	34.2	26.1–42.3	25.0
Male	17.2	10.1–24.3	7.0
Total	27.9	21.8–34	17.0

^a^ Marginal means are inflated compared to medians due to censored cases.

**Table 5:** Means and medians by BMI for survival time.

Survival (Month)	Mean ^a^	Mean CI 95%	Median
BMI at baseline (T0)			
Low	26.588	15.93–37.24	18.000
Normal	31.258	18.17–44.34	9.000
High	26.462	19.07–33.85	22.000

^a^ Marginal means are inflated compared to medians, particularly the Normal BMI because it included the majority of censored cases.

**Table 6:** Factors of survival time.

	HR (Hazards Ratio) ^a^	95% CI		Sig.
		Inferior	Superior	
Sex (female)	0.48	0.27	0.85	0.012
Hemoglobin_0	0.98	0.97	0.91	0.005
BMI_02	0.89	0.81	0.98	0.023
MUAC_02	0.90	0.79	10.0	0.172
MAMC_02	0.88	0.76	10.0	0.094

^a^ regarding death events. Hazards ratios with statistical significance (<0.05) are highlighted in bold. The Sex variable reference level was male.

## Data Availability

The data presented in this study are available upon request from the first author.
